# Human cytomegalovirus glycoprotein N polymorphisms among renal transplant recipients in India

**DOI:** 10.1186/1471-2334-14-S3-P66

**Published:** 2014-05-27

**Authors:** A Raj Kumar Patro, Lalit Dar, Sunil K Pati, Sanjay K Agarwal, Sandeep Guleria, Shobha Broor, Suresh B Boppana

**Affiliations:** 1 Departments of Microbiology, All India Institute of Medical Sciences, New Delhi, India; 2 Departments of Nephrology, All India Institute of Medical Sciences, New Delhi, India; 3 Departments of Surgery, All India Institute of Medical Sciences, New Delhi, India; 4 Department of Microbiology, All India Institute of Medical Sciences, Bhubaneswar, India; 5 Departments of Pediatrics, University of Alabama at Birmingham, Birmingham, USA; 6 Departments of Microbiology, University of Alabama at Birmingham, Birmingham, USA

## Background

Human Cytomegalovirus (HCMV) is one of the most common infectious complications in renal transplant recipients. Extensive genetic polymorphisms in envelope glycoproteins of HCMV have been demonstrated among clinical HCMV isolates. Some previous studies have proposed the potential linkage between HCMV gN genotype to the frequency of symptomatic infection and clinical outcome in renal transplant recipients. No data available on circulating HCMV strains among renal transplant recipients in India.

## Objective

To determine the cytomegalovirus strain diversity in renal transplant recipients.

## Methods

One hundred thirty two renal transplant recipients were included in this study. DNA extracted from blood samples by Qiagen kit method. Specimen DNA was amplified by PCR using glycoprotein N (*gN*) gene primers. Genotyping was carried out by RFLP and results were confirmed by direct and/or cloning followed by nucleotide sequencing of the plasmid DNA of selected strains.

## Results

HCMV detected by PCR for *gN* gene in the 83(63%) samples and sixty seven of these samples were typed by RFLP. Among these *gN1* was identified in 17(25.3 %) cases. None of the samples contained *gN2* genotype, *gN3* genotype detected in 9(13.4%) cases, while *gN4* strain was identified in 23 cases (34.3%), respectively. Infection with multiple *gN* type was seen in 18(27 %) of the samples.

## Conclusion

The results of our study showed that gN4 was the commonest gN genotype found followed by gN1. This study was supported by the Indian Council of Medical Research, Government of India.

